# Anesthetic managements, morbidities and mortalities in retroperitoneal sarcoma patients experiencing perioperative massive blood transfusion

**DOI:** 10.3389/fonc.2024.1347248

**Published:** 2024-03-05

**Authors:** Jun Wang, Jun Chen, Kunpeng Liu, Hua Zhang, Yue Wei, Libin Suo, Shuang Lan, Yanzhen Wang, Chenghua Luo, Lan Yao

**Affiliations:** ^1^ Department of Anesthesiology, Peking University International Hospital, Beijing, China; ^2^ Department of Retroperitoneal Tumor Surgery, Peking University International Hospital, Beijing, China; ^3^ Department of General Surgery, Beijing Friendship Hospital, Capital Medical University, Beijing, China; ^4^ The Clinical Epidemiology Research Center, Peking University Third Hospital, Beijing, China; ^5^ Department of Retroperitoneal Tumor Surgery, Peking University People’s Hospital, Beijing, China

**Keywords:** retroperitoneal sarcoma, massive blood transfusion, anesthesia management, morbidity, mortality

## Abstract

**Objective:**

Given high risks of major bleeding during retroperitoneal sarcoma(RPS) surgeries, severe complications and deaths are common to see perioperatively. Thus, effective anesthetic management is the key point to ensuring the safety of patients. This study aimed to introduce anesthesia management and mortalities in RPS patients receiving massive blood transfusions during surgeries.

**Methods:**

Records of RPS surgeries under general anesthesia from January 2016 through December 2021 were retrospectively retrieved from our database. Patients who received massive blood transfusions (MBT) exceeding 20 units in 24h duration of operations were finally included in this study. Demographics, modalities of anesthesia management, blood loss, transfusion, peri-anesthesia biochemical tests as well as morbidities and mortalities were collected. Risk factors of postoperative 60d mortality were determined through logistic regression in uni-and multi-variety analysis using the statistics software STATA 17.0.

**Results:**

A total of 70 patients (male 31) were included. The mean age was 50.1 ± 15.8 years. All patients received combined resections of sarcoma with involved organs under general anesthesia. Mean operation time and anesthesia time were 491.7 ± 131.1mins and 553.9 ± 132.6mins, respectively. The median intraoperative blood loss was 7000ml (IQR 5500,10000ml). Median red blood cells (RBC) and fresh frozen plasma (FFP) transfusion were 25.3u (IQR 20,28u), and 2400ml (IQR 2000,3000ml), respectively. Other blood products infusions included prothrombin complex concentrate (PCCs), fibrinogen concentrate (FC), platelet(plt) and albumin(alb) in 82.9% (58/70), 88.6% (62/70), 81.4% (57/70) and 12.9% (9/70) of patients. The postoperative severe complication rate(Clavien-Dindo grade≥3a) was 35.7%(25/70). A total of 7 patients (10%) died during the postoperative 60-day period. BMI, volumes of crystalloid infusion in anesthesia, and hemoglobin and lactate levels at the termination of operation were found significantly associated with postoperative occurrence of death in univariate analysis. In logistic multivariate analysis, extended anesthesia duration was found associated with postoperative venous thrombosis embolism (VTE) and severe complication. The lactate level at the immediate termination of the operation was the only risk factor related to perioperative death (p<0.05).

**Conclusion:**

RPS patients who endure MBT in surgeries face higher risks of death postoperatively, which needs precise and effective anesthesia management in high-volume RPS centers. Increased blood lactate levels might be predictors of postoperative deaths which should be noted.

## Introduction

Retroperitoneal sarcoma (RPS) represents a heterogeneous group of malignant mesenchymal neoplasms with an annual incidence of less than 0.5 per 100,000 population (1, 2). RPS often presents as an enlarged mass involving multiple organs or major vessels. Surgery remains the mainstay treatment for localized RPS (3, 4). An extended resection of the sarcoma with adherent structures and organs has been introduced and advocated for achieving better local control and longer overall survival (5, 6). However, this approach poses significant challenges for surgeons and anesthesiologists due to the deep anatomical sites, wide excisions, and intraoperative hemodynamic instabilities (7, 8). Perioperative major bleeding in RPS surgery has been reported as the leading cause and independent factor associated with surgical mortality (9, 10). Meanwhile, major bleeding that occurs intraoperatively remains a significant challenge to handle, not only for surgeons but also for anesthesiologists. Intraoperative blood loss (IBL) exceeding 2000ml, followed by massive blood transfusion (MBT), can result in a significant increase in severe complications such as coagulation disorders, secondary hemorrhages, renal dysfunctions, thrombosis, cardiovascular events, and more (11). Nevertheless, blood transfusion was also reported as an independent risk factor associated with severe complications either in primary or recurrent RPS surgeries, according to the largest case series of 1007 cases from Transatlantic Australasian RPS Working Group (TARPSWG) (10, 12). To ensure the safety of RPS surgeries, hybrid surgical techniques are key. Nevertheless, proactive and effective anesthetic managements, applied with a damage-control principle also plays an utmost important role. Immediate hemorrhage control, limited intravenous crystalloid, early administration of warmed blood products, balanced massive transfusion, and permissive hypotension are the mainstay approaches for managing hemorrhage in surgical patients. Appropriate components and ratios of blood products have been reported to be associated with lower 24-hour mortality in massively transfused patients (13–15). However, to date, no data have been reported on the anesthetic strategies and related outcomes in the RPS area. This study was conducted at a high-volume RPS referral center in China, which performs over 400 surgeries annually for retroperitoneal tumors. The study aims to introduce the anesthetic management and experiences, and analyze the risk factors associated with postoperative morbidity and mortality.

## Patients and methods

### Study design and patients

It was a retrospective study, conducted in accordance with the Declaration of Helsinki (as revised in 2013), following the STROBE retrospective cohort guideline. It was approved by the institutional review board of Peking University International Hospital (PKUIH-NO.2022-KY-0032-01) and individual consent for this retrospective analysis was waived. Medical records of consecutive patients undergoing resection surgeries under general anesthesia for RPS at PKUIH from January 2016 through December 2021 were retrieved from our prospectively collected database. Patients who received transfusion ≥20 units of packed red blood cells (RBCs) in peri-operative 24 hours were defined as MBT and included in this study. Patients with histological subtypes of retroperitoneal tumors other than RPS were excluded from this study. Patients who underwent tumor biopsy through laparotomy only instead of radical resections were also excluded. Data collection included demographics, perioperative variables, detailed anesthesia managements with intraoperative dynamic blood cell and biochemical tests, operative outcomes as well as postoperative 60-d survivals.

### Surgical and anesthesia protocols

All surgeries were performed by the same surgical team after careful MDT discussion. The extended resection policy was applied in most patients with localized or uni-focal tumors. Major vessels involved by tumors were often isolated, repaired, or transected/ligated with or without reconstruction. Decisions were made according to the surgeons’ discretion. Patients with metastasis, multifocal tumors or obstructive symptoms in the digestive tract or urinal tract were also operated under the palliative surgical policy. A novelty ipsilateral lipectomy was performed in patients with primary retroperitoneal liposarcoma in order to decrease the localized recurrence rate.

All patients were treated with general anesthesia through endotracheal intubation. Central and peripheral venous accesses were built for infusions. Continuous infusion of Remifentanil, Propofol and Dexmedetomidine with occasional inhalation of Sevoflurane or Desflurane were normally used for maintaining anesthesia effects. Speeding bolus or transfusion of blood products, crystalloids with vasoactive agents such as norepinephrine were administered when dealing with intraoperative major bleeding. Recording the amounts of IBL, blood components and crystalloid infusions as well as values of blood glucose, creatinine, and blood gas analysis at the entry and exit of the operating room (OR) was also the routine process of anesthesia management.

### Statistics

Categorical variables were reported as frequency (%) and continuous variables were reported as mean with standard deviation (SD) or median with interquartile range (IQR). Morbidities were evaluated by Clavien-Dindo grading system. Logistic regression was used to determine risk factors of postoperative VTE and deaths in univariate and multivariate analysis. Variables acquired P value less than 0.1 in univariate analysis were included and calculated in the multivariate analytic equation. A P value of less than.05 was considered statistically significant. The software of STATA Version 17.0 was used for statistics.

## Results

### Patient characteristics

**Figure 1:**
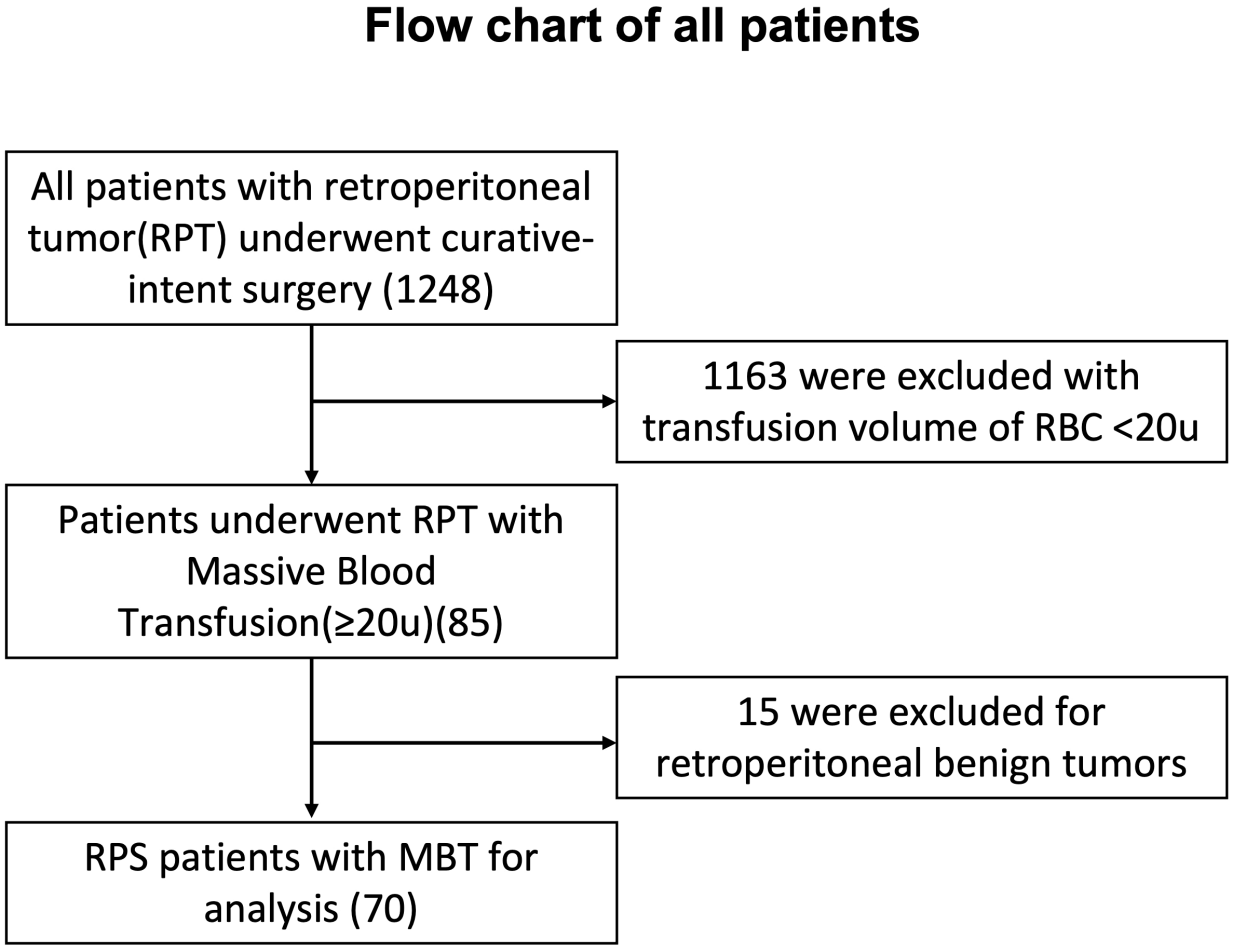
Flow chart of all patients with retroperitoneal tumor underwent surgery.

There were 70 RPS patients (male 39) experiencing MBT over 20u in peri-operative 24 hours finally included in this study. The flow chart was introduced in **Figure 1**. The mean age was 50.1 ± 15.8 years old. Seventeen out of 70 (24.3%) patients were primary RPS cases, while other 53 patients were recurrent RPS, including 33 (47.1%) patients who had over 3 times of histories of surgeries. Preoperatively, 37.1% (26/70) of patients had miscellaneous comorbidities, consisting 8 diabetes, 7 hypertensions and 7 cardiovascular diseases. Patients’ characteristics were described in **Table 1**.

**Table 1:** Characteristics of all patients experiencing MBT≥20u in peri-operative 24 hours.

Variables	N	%
Total	70	100
Age(mean±SD)	50.1 ± 15.8	–
Male gender	39	55.7
BMI (mean±SD)	22.2±3.2	–
RPS		
Primary	17	24.3
Recurrent	53	45.7
2^nd^ recurrence	20	28.6
≥3^rd^ recurrence	33	47.1
Comorbidities		
Diabetes	8	11.4
Hypertension	6	8.6
Cardiovascular disease	6	8.6
Anemia	6	8.6
Renal Dysfunction	3	4.3
Venous Thrombosis	3	4.3
RPS subtypes		
Liposarcoma	42	60
Leiomyosarcoma	4	5.7
Solitary fibrous tumor	11	15.7
Other	13	18.6

### Anesthesia and operative outcomes

Preoperatively, the general health status of all patients was assessed using the ASA classification system, resulting in the following distribution: 4 patients were classified as ASA I, 30 as ASA II, and 36 as ASA III. No patients were classified as ASA IV. All patients received combined resections of sarcoma with involved organs under general anesthesia. A total of 99 organs or major vessels were resected in 70 surgeries, with 12 surgeries combining resection of three or more organs and five surgeries with major vessel resections, either with or without reconstructions. The average operation and anesthesia durations were 491.7 ± 131.1mins and 553.9 ± 132.6mins, respectively. The median intraoperative blood loss was 7000ml (interquartile range [IQR] 5500,10000), with 19 cases (27.1%) exceeding 10000ml. **Table 2** showed the details of anesthesia and operative outcomes.

**Table 2:** Anesthesia and operative outcomes of all subjects.

Variables	N	%
Total	70	100
ASA Score		
1	4	5.7
2	30	42.9
3	36	51.4
Operation time(mean ± SD, min)	491.7 ± 131.1	–
Anesthesia time(mean ± SD, min)	553.9 ± 132.6	–
Blood loss (median, IQR)(ml)	7000(5500,10000)	–
Patients No underwent organs resection	46	65.7
1	14	20.0
2	20	28.6
≥3	12	17.1
Organs resected in all patients		
Stomach & Duodenum	6	8.6
Intestine	12	17.1
Colon & Rectum	26	37.1
Kidney	15	21.4
Ureter	6	8.6
Spleen & Pancreas	9	12.9
Liver	3	4.3
Major Vessels resected in all patients		
Inferior Vena Cava	3	4.3
External Iliac Vein	1	1.4
External Iliac Artery	2	2.8
Morbidities	46	65.7
Venous thrombosis	16	22.9
Acute renal dysfunction	13	18.6
Infectious	9	12.9
Acute lung Injury	7	10.0
ARDS	6	8.6
Miscellaneous fistular (intestine, urinary, gastric)	12	17.1
Cardiac events	4	5.7
Complication Classification		
I-II	21	30.0
III-IV	18	25.7
V(Death)	7	10.0
Postoperative Day in hospital (median, IQR)	29.5(20,44)	–

### Infusions in anesthesia

All patients received transfusions of blood products and fluids, as well as vasoactive medications such as norepinephrine, dopamine, or epinephrine, to maintain stable circulation during anesthesia (**Table 3**). The median red blood cell (RBC) transfusion volume was 25.3 units (IQR 20, 28), and fresh frozen plasma(FFP) transfusion volume was 2400 ml (IQR 2000, 3000). Prothrombin complex concentrate (PCCs), fibrinogen concentrate(FC), platelets, and albumin were administered to 82.9% (58/70), 88.6% (62/70), 81.4% (57/70), and 12.9% (9/70) of patients, respectively. Additionally, crystalloid and artificial colloid fluids were transfused with a median volume of 6200 ml (IQR 4000, 7450) and 2250 ml (IQR 1500, 3000), respectively. In the postoperative 24-hour period, 39 patients received additional RBC transfusions, and 47 patients received additional plasma infusions.

**Table 3:** Details of infusions during anesthesia in all MBT patients.

Infusions	N (%)	Median	InterQual Range
RBC(unit)	70(100)	22	22,28
FFP(ml)	70(100)	2400	2000,3000
PCCs(ml)	58(82.9)	600	400,1200
FC(unit)	62(88.6)	1.5	1,2
Platelet	9(12.9)	0	0,0
Albumin(g)	57(81.4)	400	150,500
Crystalloid(ml)	70(100)	6200	4000,7450
Artificial colloid fluids(ml)	70(100)	2250	1500,3000

### Blood tests in anesthesia

During the entire anesthesia procedure, comprehensive assessments such as complete blood count, blood chemistry analysis, coagulation profiling, and arterial blood gas analysis were conducted for all patients. These tests encompass the preoperative, intraoperative, and postoperative phases, spanning a duration of 24 hours. Details were listed in **Table 4**.

**Table 4:** Blood tests in different time phases of anesthesia.

Tests	Values		P Value
	Median/Mean	IQR/ ± SD	
Pre-anesthesia term			
Hemoglobin(g/dl)	110.0	17.2	
Lactate(mmol/l)	0.8	0.6, 0.9	
Platelet(10^9/L)	260.5	200, 368	
Albumin(g)	34.6	5.0	
Fibrinogen(mg/dl)	399	325,459	
PT(s)	11.7	11.2, 12.9	
APTT(s)	30.73	3.35	
INR	1.1	1,1.2	
FDP	4.4	2.7, 8.4	
D-Dimer	429	238, 776	
Creatine	61	51, 89	
Terminal time of anesthesia*			
Hemoglobin(g/dl)	88	20.8	0.000
Lactate(mmol/l)	2	1.1, 3.9	0.000
24-hours post anesthesia*			
Hemoglobin(g/dl)	93.8	26.9	0.000
Platelet(10^9/L)	66	46, 110	0.000
Albumin(g)	29.4	7.0	0.210
Fibrinogen(mg/dl)	217.5	192,279.5	0.000
PT(s)	12.95	12.1, 14.2	0.004
APTT(s)	31.4	28.8, 36.6	0.015
INR	1.2	1.1, 1.3	0.002
FDP	7.3	3.92, 14	0.15
D-Dimer	734	392, 1545	0.185
Creatine	80	57, 102	0.991

*Compared with correspondent variables in pre-anesthesia term.

### Morbidities and mortalities

Postoperatively, a total of 52 patients (74.3%) were admitted to the Intensive Care Unit (ICU), and they required a median duration of mechanical ventilation of 3.9 days. The overall postoperative complication rate was 65.7% (46/70) with 35.7%(25/70) of patients experiencing severe morbidities(Clavien-Dindo grade≥3a). Anesthesia duration was the only risk factors associated to postoperative severe morbidity in logistic multivariate analysis, though there were lots of anesthesia related factors associated with severe morbidity in univariate analysis, like infusion volume of RBC and FFP, intraoperative blood loss, hemoglobin and lactate level at the termination of operation, etc. (**Table 5**). The most common complications observed were venous thrombosis (16 cases) and acute renal dysfunction (13 cases). Three patients underwent salvage reoperations due to hemostasis (2 cases) and intestinal fistula (1 case). During the postoperative 60-day period, there were 7(10%) patients deceased. The main causes of death included 1 immediate intraoperative bleeding, 4 delayed bleeding, and 2 septic shocks with severe abdominal infections. In univariate analysis, durations of operation and anesthesia, and repairing/resection of major vessels were significant related to postoperative onset of VTE (**Table 6**). Factors such as body mass index (BMI), volumes of crystalloid infusion during anesthesia, and hemoglobin and lactate levels at the termination of operation were significantly associated with postoperative mortality. However, in logistic multivariate analysis, lactate level at the termination the operation was identified as the only risk factor related to perioperative mortality. The anesthesia time and was found significant associated to postoperative VTE (p<0.05) (**Table 6**).

**Table 5:** Univariate and multivariate analysis of postoperative severe morbidities in all patients.

Variables	N	Severe Morbidities		Uni-variate	Multivariate		
		Yes	No	P	OR	95% CI	P
Total	70	25	45				
Age(mean±SD)	50.1 ± 15.8			0.618			
Male gender	31	12	19	0.641			
BMI(mean±SD)	22.2±3.2			0.704			
ASA Score							
1	4	1	3	0.569			
2	30	10	20				
3	36	14	22				
Comorbidities							
Diabetes	8	3	5	0.911			
Hypertension	6	1	5	0.329			
Cardiovascular disease	6	4	2	0.120			
Preoperative blood tests							
Hb (mean ± SD, g/l)	110.0 ± 11.2			0.652			
Alb(median, g/l)	34.1			0.673			
Plt(median, 10^9/l)	260.5			0.797			
Fg(median, mg/dl)	399			0.529			
PT(median, s)	11.7			0.464			
APTT(mean ± SD, s)	30.73±3.35			0.064	1.193	0.980-1.453	0.079
INR(median)	1.1			0.844			
FDP(median, mg/l)	4.4			0.222			
D-dimer(median, mg/l)	429			0.293			
Anesthesia time(mean ± SD, min)	553.9 ± 132.6			0.034	1.007	1.000-1.012	***0.025***
Repairing/Resection of major vessels during surgeries	12	6	6	0.637			
Organ resections							
No	24	7	17	0.410			
Yes	46	18	28				
Blood tests at the end of operation							
Hb (mean ± SD, g/l)	88.0 ± 20.8			0.004	0.973	0.935-1.013	0.181
Lactate(median, mmol/l)	2.0			0.033	1.060	0.785-1.431	0.703
IBL(median, ml)	7000			0.015	1.000	1.000-1.000	0.903
Blood products infusions during operation							
RBC(median. U)	22			0.012	1.053	0.888-1.250	0.551
FFP(median, ml)	2400			0.049	1.000	0.999-1.000	0.605
PCC(median, u)	600			0.124			
FC(median, mg/dl)	1.5			0.103			
PLT(median, range, u)	0(1-10)			0.063	4.101	0.605-27.80	0.148
Crystalloid infusion during anesthesia(mean ± SD, ml)	6254.8 ± 1923.5			0.054	1.000	1.000-1.000	0.971
Colloid infusion during anesthesia(median, ml)	2250			0.072	1.000	1.000-1.000	0.465

**Table 6:** Univariate and multivariate analysis of postoperative VTE in all patients.

Variables	N	VTE		Uni- variate	Multivariate		
		+	-	P	OR	95% CI	P
Total	70	16	54				
Age(mean±SD)	50.1 ± 15.8			0.193			
Male gender	31	2	29	0.409			
BMI(mean±SD)	22.2±3.2			0.489			
ASA Score							
1	4	1	3	0.903			
2	30	8	22				
3	36	8	28				
Comorbidities							
Diabetes	8	2	6	0.960			
Hypertension	6	2	4	0.589			
Cardiovascular disease	6	2	4	0.589			
Preoperative blood tests							
Hb (mean ± SD, g/l)	110.0 ± 11.2			0.704			
Alb(median, g/l)	34.1			0.365			
Plt(median, 10^9/l)	260.5			0.726			
Fg(median, mg/dl)	399			0.946			
PT(median, s)	11.7			0.214			
APTT(mean ± SD, s)	30.73±3.35			0.476			
INR(median)	1.1			0.127			
FDP(median, mg/l)	4.4			0.568			
D-dimer(median, mg/l)	429			0.414			
Operative time(mean ± SD, min)	491.7 ± 131.1			0.004	0.978	0.956,1.000	0.060
Anesthesia time(mean ± SD, min)	553.9 ± 132.6			0.001	1.031	1.007,1.058	***0.013***
Repairing/Resection of major vessels during surgeries	12	6	6	0.022	5.053	1.066,23.964	***0.041***
Use of Tranexamic acid during anesthesia	40	10	30	0.872			
Blood tests at the end of operation							
Hb (mean ± SD, g/l)	88.0 ± 20.8			0.430			
Lactate(median, mmol/l)	2.0			0.691			
IBL(median, ml)	7000			0.543			
Blood products infusions during operation							
RBC(median. U)	22			0.222			
FFP(median, ml)	2400			0.436			
PCC(median, u)	600			0.778			
FC(median, mg/dl)	1.5			0.831			
PLT(median, range, u)	0(1-10)			0.578			
Crystalloid infusion during anesthesia(mean ± SD, ml)	6254.8 ± 1923.5			0.248			
Colloid infusion during anesthesia(median, ml)	2250			0.191			
Total RBC infusion within 24h	24			0.224			
Total plasma infusion within 24h	2800			0.295			

## Discussion

Patients with retroperitoneal tumors often present with large tumor sizes and involvement of surrounding blood vessels and organs, which increases the difficulty of surgical treatment. Prolonged compression of the retroperitoneal space by the tumor can lead to adhesion formation between the tumor and vascular walls, resulting in vascular occlusion and compensatory formation of extensive collateral circulation vessels supplying the tumor (16, 17). Besides, extended surgical resection with adjacent/infiltrated major vessels and organs in order to achieve better local controls also poses challenges for intraoperative bleeding control, and significant intraoperative bleeding frequently occurs (7, 18). In this study, an average of 1.5 organs were resected in whole patients, including 12 patients with 3 or more organs resected and 5 patients with major vessel resections. The intraoperative blood loss ranged from 3000ml to a devastated volume of 25800 ml. Therefore, sufficient blood supply, precise and efficient anesthesia managements should be administered perioperatively, especially for those elderly, multiple comorbidities or recurrent cases with multiple surgical histories (19).

Throughout the entire anesthesia process, goal-directed fluid treatment has been strongly recommended and implemented using various hemodynamic monitoring methods, including arterial pressure or central venous pressure monitoring, pulse contour analysis, and transesophageal echocardiography (20, 21). Instead of crystalloid resuscitation, damage control resuscitation with balanced components in a 1:1:1 fashion or whole blood (WB) has been advocated as a crucial component in the resuscitation of major bleeding (22–24). Transfusion of FFP, platelets, and RBC at a higher 1:1:1 ratio, compared to the conventional 1:1:2 ratio, is associated with a lower incidence of complications and mortality in patients with severe trauma (15, 25, 26). In this particular study, the median transfusion volume of FFP and RBC was 2573ml and 25.2 units, resulting in a ratio of 1:1. Based on this fluid treatment approach, the case series observed a severe morbidity rate of 35.7% and a 30-day mortality rate of 7%, which is notably lower than the reported ranges of 20% to 60% in patients with acute trauma (22, 27). In addition, whole blood (WB) has been considered as an ideal and beneficial option for patients undergoing massive blood transfusion (MBT). However, one of the challenges associated with WB transfusion is the time required to conduct safety tests on the blood, which can lead to significant depletion of coagulation factors. Otherwise, WB transfusion was also associated with higher platelet-to-red blood cell (PLT : RBC) and plasma-to-red blood cell (plasma:RBC) ratios, which warrants further discussion and investigation (28).

In situations of significant blood loss, the depletion of blood components is not uniform. The concentrations of coagulation factors are insufficient to adequately increase or maintain the already low plasma concentrations in bleeding patients. The median fibrinogen level was dropped with PT and APTT extended significantly from preoperative to postoperative term(p<0.05). Studies on surgical patients receiving massive blood transfusions have also shown that higher fibrinogen levels at the end of surgery are associated with increased patient survival rates. However, administration of PCC or FC was reported associated with an increased risk of thrombotic events in trauma patients according to several earlier studies (29, 30). On the opposite, Florian et al. discovered that 68% out of 1630 patients experiencing severe hemorrhage at 6h and 72% at 24h co-administration of FFP and FC in that complied with recommendations, mortality was systematically lower than expected in contrast to non-compliant without FFP and FC using subgroups (31). In this study, 82.9% and 88.6% out of 70 patients underwent PCC and FC transfusions respectively. Instead of the PCC and FC using, the anesthesia duration was the only risk factors found significantly associated to VTE and severe morbidity (P<0.05) (**Table 6**, **7**). Thus, whether PCC/FC administration in patients with MBT remains further exploration through high-quality data. Point of Care Testing (POCT), Rotational thromboelastometry (ROTEM), thrombelastography (TEG), might be useful tools recommended in monitoring the real-time deficiencies of concentrates of blood cells and coagulation factors, decreasing transfusion related complications and mortalities (32–34). Therefore, individual use of laboratory test-based approach with coagulation factor concentrates is essential for fast and goal-directed therapy to address bleeding-induced coagulation factor deficiency (35).

**Table 7:** Univariate and multivariate analysis of postoperative deaths in all patients.

Variables	N	Death		Uni- variate	Multivariate		
		+	-	P	OR	95% CI	P
Total	70	7	63				
Age(mean±SD)	50.1 ± 15.8			0.263			
Male gender	31	2	29	0.452			
BMI(mean±SD)	22.2±3.2			0.032	0.830	0.464,1.488	0.533
ASA Score							
1	4	1	3	0.404			
2	30	2	28				
3	36	4	32				
Preoperative blood tests							
Hb (mean ± SD, g/l)	110.0 ± 11.2			0.883			
Alb(median, g/l)	34.1			0.857			
Plt(median, 10^9/l)	260.5			0.252			
FC(median, mg/dl)	399			0.368			
PT(median, s)	11.7			0.213			
APTT(mean ± SD, s)	30.73±3.35			0.074	1.852	0.981,3.495	0.057
INR(median)	1.1			0.216			
FDP(median, mg/l)	4.4			0.941			
D-dimer(median, mg/l)	429			0.583			
Lactate(median, mmol/l)	0.8			0.085	1.491	0.588,3.776	0.400
Glucose(median,mmol/l)	5.2			0.229			
Operative time(mean ± SD, min)	491.7 ± 131.1			0.857			
Anesthesia time(mean ± SD, min)	553.9 ± 132.6			0.724			
Organ resections during surgeries							
0	24	1	23	0.399			
1	14	1	13				
≥2	32	5	27				
Repairing/Resection of major vessels during surgeries	12	2	10	0.595			
Blood tests at the end of operation							
Hb (mean ± SD, g/l)	88.0 ± 20.8			0.010	0.988	0.891-1.096	0.823
Lactate(median, mmol/l)	2.0			0.002	2.401	1.021-5.651	***0.045***
Glucose(median,mmol/l)	10.2			0.558			
IBL(median, ml)	7000			0.095	1.000	1.000-1.000	0.775
Blood products infusions during operation							
RBC(median. U)	22			0.138			
Plasma(median, ml)	2400			0.499			
PCC(median, u)	600			0.085	1.001	0.998-1.005	0.475
FC(median, mg/dl)	1.5			0.065	0.544	0.078-3.793	0.539
Alb(median, g)	300			0.109			
Plt(median, range, u)	0(1-10)			0.252			
Crystalloid infusion during anesthesia(mean ± SD, ml)	6254.8 ± 1923.5			0.031	1.000	0.999-1.000	0.896
Colloid infusion during anesthesia(median, ml)	2250			0.926			
Total RBC infusion within 24h	24			0.063	0.979	0.824-1.163	0.807
Total plasma infusion within 24h	2800			0.380			

ASA, American Society of Anesthesia; RBC, Red blood cell; Hb, Hemoglobin; Alb, Albumin; Plt, Platelet; PCC, Prothrombin complex concentrate; FC, Fibrinogen concentrate; PT, Prothrombin time; APTT, Activated partial thromboplastin time; INR, International normalized ratio; FDP, Fibrin degradation product; IBL, Intraoperative blood loss.

RPS surgeries pose persistent challenges for surgeons and anesthesiologists, carrying elevated risks of severe postoperative complications and mortalities, despite the implementation of comprehensive management strategies. The need for transfusion of blood products during RPS surgeries has been a significant predictor of severe postoperative adverse events in previous reports from TARPSWG (10, 12). However, in our study, statistical significance was not achieved. Notably, only anesthesia durations were found to be associated with postoperative severe complications. This finding may be attributed to the case selection in our study, which focused on patients in extremely emergent situations requiring major transfusions, excluding those who did not require blood product transfusions. Owing to the extensive volume of retroperitoneal sarcoma (RPS) surgical experiences, coupled with the meticulous application of surgical and anesthesiologic interventions, as well as sustained postoperative blood transfusions, postoperative bleeding did not emerge as a primary complication, contrary to previous cases reported by TARPSWG. Conversely, venous thrombosis emerged as the most prevalent adverse event after massive blood transfusion (MBT), potentially associated with prolonged anesthesiologic durations. In the examination of mortality factors, the singular factor correlated with postoperative 60-day death was lactate levels. Elevated lactate levels have been consistently reported as a robust risk factor linked to increased mortality in patients experiencing septic shock, cardiac arrest, hemorrhagic shock, and major surgeries (36–39). A lactate level of ≥2.5 mmol/L has been identified as the optimal threshold for predicting 28-day mortality in severe sepsis and septic shock patients (40). In our study, the lactate levels in the seven deceased patients rose to an average of 6.01 mmol/L at the conclusion of surgeries, compared to the preoperative mean value of 2.26 mmol/L(p<0.001). This value of 6.01 mmol/L was also significantly higher than the level of 2.55 mmol/L in the other 63 surviving patients at the same juncture, indicating a substantial increase in postoperative mortality.

This study has several intrinsic limitations. Due to the rarity and unavailability of platelet, the ratio of FFP: PLT: RBC were not fully compliant to recommendations according to some MBT guidelines and protocols. The exact effects of more PLT application in perioperative mortality and VTE need further exploration. This study is a retrospective analysis with limited numbers of subjects. And the severity and complexity of the RPS remains diverse. Selective bias was not avoidable as no significant statistical relationship were found between fluid/blood product infusion and postoperative morbidities and mortalities.

In general, precisive and effective anesthesia managements using goal-directed and blood test guided strategy during RPS surgeries are utmost important which may arrive at acceptable postoperative morbidities and mortalities in MBT patients. Patients with higher lactate level at the termination of operation with extended anesthesia duration deemed to have higher possibilities of postoperative VTE and deaths which should be alerted.

## Data availability statement

The original contributions presented in the study are included in the article/supplementary material. Further inquiries can be directed to the corresponding authors.

## Ethics statement

The studies involving humans were approved by Peking University International Hospital Institutional Review Board. The studies were conducted in accordance with the local legislation and institutional requirements. The ethics committee/institutional review board waived the requirement of written informed consent for participation from the participants or the participants’ legal guardians/next of kin because It was a retrospective study without any interventions of the subjects.

## Author contributions

JW: Data curation, Project administration, Writing – original draft. JC: Funding acquisition, Methodology, Resources, Writing – review & editing, Writing – original draft, Formal analysis. KL: Data curation, Investigation, Validation, Writing – review & editing. HZ: Formal analysis, Software, Writing – original draft. YWe: Data curation, Methodology, Writing – original draft. LS: Data curation, Methodology, Writing – original draft. SL: Data curation, Methodology, Writing – original draft. YWa: Data curation, Methodology, Writing – original draft. CL: Resources, Supervision, Validation, Writing – review and editing. LY: Investigation, Project administration, Supervision, Validation, Writing – review & editing.
